# Evaluation of visual food stimuli paradigms on healthy adolescents for future use in fMRI studies in anorexia nervosa

**DOI:** 10.1186/s40337-023-00761-8

**Published:** 2023-03-06

**Authors:** Agnieszka Dąbkowska-Mika, Ruth Steiger, Manuela Gander, Nina Haid-Stecher, Martin Fuchs, Kathrin Sevecke, Elke Ruth Gizewski

**Affiliations:** 1 Department of Neuroradiology, Medical University of Innsbruck, Anichstrasse 35, 6020 Innsbruck, Austria; 2 Neuroimaging Research Core Facility, Medical University of Innsbruck, Anichstrasse 35, 6020 Innsbruck, Austria; 3 Department of Child and Adolescent Psychiatry, Psychotherapy and Psychosomatics, Tirol Kliniken, Milserstrasse 10, 6060 Hall in Tirol, Austria

**Keywords:** Evaluation of visual food stimulus, High-calorie and low-calorie food images, Study paradigm optimization, Functional MRI

## Abstract

**Background:**

Mostly, visual food stimuli paradigms for functional Magnetic Resonance Imaging are used in studies of eating disorders. However, the optimal contrasts and presentation modes are still under discussion. Therefore, we aimed to create and analyse a visual stimulation paradigm with defined contrast.

**Methods:**

In this prospective study, a block-design fMRI paradigm with conditions of randomly altering blocks of high- and low-calorie food images and images of fixation cross was established. Food pictures were rated in advance by a group of patients diagnosed with anorexia nervosa to address the dedicated perception of patients with eating disorders. To optimize the scanning procedure and fMRI contrasts we have analysed neural activity differences between high-calorie stimuli versus baseline (*H *vs.* X*), low-calorie stimuli versus baseline (*L *vs.* X*) and high- versus low-calorie stimuli (*H *vs.* L*).

**Results:**

By employing the developed paradigm, we were able to obtain results comparable to other studies and analysed them with different contrasts. Implementation of the contrast *H *versus* X* led to increased blood-oxygen-level-dependent signal (BOLD) mainly in unspecific areas, such as the visual cortex, the Broca´s area, bilaterally in the premotor cortex and the supplementary motor area, but also in thalami, insulae, the right dorsolateral prefrontal cortex, the left amygdala, the left putamen (*p* < .05). When applying the contrast *L *versus* X*, an enhancement of the BOLD signal was detected similarly within the visual area, the right temporal pole, the right precentral gyrus, Broca´s area, left insula, left hippocampus, the left parahippocampal gyrus, bilaterally premotor cortex and thalami (*p* < .05). Comparison of brain reactions regarding visual stimuli (high- versus low-calorie food), assumed to be more relevant in eating disorders, resulted in bilateral enhancement of the BOLD signal in primary, secondary and associative visual cortex (including fusiform gyri), as well as angular gyri (*p* < .05).

**Conclusions:**

A carefully designed paradigm, based on the subject’s characteristics, can increase the reliability of the fMRI study, and may reveal specific brain activations elicited by this custom-built stimuli. However, a putative disadvantage of implementing the contrast of high- versus low-calorie stimuli might be the omission of some interesting outcomes due to lower statistical power.

*Trial registration* NCT02980120.

**Supplementary Information:**

The online version contains supplementary material available at 10.1186/s40337-023-00761-8.

## Abstract (summary)

Although the relationship with food is crucial for living, its underlying mechanisms (e.g., neurological, cognitive, physiological) are still not fully discovered. The development of functional magnetic resonance imaging made it possible to explore brain’s responses to images of food. However, a proper methodological analysis of the research paradigm is still lacking. Here, we present the optimization of visual food stimuli paradigms achieved by comparison of neural activations of 20 female healthy adolescents after applying particular contrasts (i.e., high- versus low-calorie food images, high-calorie food images versus baseline, low-calorie food images versus baseline). Application of the contrast high- versus low-calorie food images resulted in stronger neural activation in visual cortex (including fusiform gyri) and angular gyri. This study highlights the importance of choosing a proper contrast regarding the study hypothesis, as it may induce more specific results. However, it may lead to loss of some outcomes, due to lower statistical power. Additionally, we have performed an evaluation of visual food stimuli chosen by patients diagnosed with anorexia nervosa. They have selected images of the most and the least willingly eaten meals. Although they didn’t know the exact calorie content, they chose intuitively photos later classified as extremely high- or low-caloric.

**Supplementary Information:**

The online version contains supplementary material available at 10.1186/s40337-023-00761-8.

## Background

According to Maslow, the need for food is one of the elemental needs, at the base of the iconic pyramid [1]. However, the basicity of this need does not necessarily lead to an appropriate relationship with food. Nowadays, the majority of inhabitants of Western countries don´t face famine. In contrary, access to an excessive amount of food, which is often unhealthy, is problematic. More than 65% of adults are overweighed or obese in United States of America. Even 31% of children (6–19 years old) stay at risk of overweight or they are already overweighed [2]. Prevalence of obesity in children has been spreading rapidly in last decades [3].

The same tendency is recorded in eating disorders (ED) [4], whereas the typical onset of ED is in puberty [5–7]. The highest mortality rate among ED (and all psychiatric diseases in total) occurs in anorexia nervosa [8]. Anorexia nervosa (AN) is a major psychiatric disorder, characterized as restriction in calorie intake (what usually lead to low body mass), intense fear of gaining weight and distorted body image [5].

Only in-depth understanding of the response to food stimuli in healthy people enables a thorough recognition of the pathophysiology of eating disorders and consequently the right therapy. There are still inconclusive results regarding the reaction towards visual food stimuli during fasting in healthy subjects due to their age. There are studies which found similarity across age groups [9], and quite contrary, there are studies which present that some differences may occur due to maturation [10, 11]. Nevertheless, teenagers can be considered to be more sensitive to high-calorie food, and unhealthy food visual stimuli are more motivating and rewarding for children than for adults, as shown in van Meer et al. [12], where children express greater activation in the left precentral gyrus.

Up-to-date, few standardized datasets with food images are available, and most of them have been published recently [13–15]. The biggest advantage of them is the possibility of easy comparison among different studies. On the other hand, a self-established base set of pictures could more accurately be accommodated in the design, considering special issues (e.g., religious background or cultural context) and special perceptions in patients with eating disorders [16].

In order to obtain the reliability of our experiment, we decided to adjust stimuli according characteristics of the local participants by creating our own picture database, with the main focus on classifying the stimuli regarding either high- or low-calorie content. Similar to other studies dealing with eating disorders, which also included control groups [17–26], we analysed our design within a fMRI study exerted on healthy young volunteers. Typically, the research on eating disorders focuses on young females as they make up the majority of patients [27]—to facilitate later comparisons we made sure that the group of healthy participants was homogeneous in age and sex.

### The aim of the study

The primary focus of the current study was to optimize the scanning procedure and fMRI contrast of food images. We aimed to analyse neural activity differences between high-calorie stimuli versus baseline (*H *vs.* X*), low-calorie stimuli versus baseline (*L *vs.* X*) and high- versus low-calorie stimuli (*H *vs.* L*). Our hypothesis was, that a paradigm incorporating food images that were either classified as low- or high-calorie images, and analysed with the contrast *H *versus* L*, will create an enhanced BOLD signal in gray matter that is typically associated with viewing high-calorie food images, distinguishing it from cerebral response to low-calorie food pictures. Secondly, we assumed that this contrast will be more suitable for studies investigating eating disorders, and is also applicable on healthy volunteers. Based on previous studies, we hypothesized that a given contrast would evoke greater activation within the fusiform gyrus, frontal area, insula and higher order of visual cortex as these regions are linked to processing food information and vision [28–32]. To our knowledge, there are few published findings regarding the evaluation of food stimuli [19], but none included ratings conducted by the target group for a future survey of eating disorders, especially anorexia nervosa. We have intended to create a dataset of visual stimuli, which would be tailored for local healthy participants, and be employed for future applications in eating disorder studies. The ethical approval of the Ethics Committee of the Medical University of Innsbruck and a lawful, informed consent from each participant was obtained prior to the start of the study.

## Methods

### Characteristic of the participants

23 female healthy adolescents were recruited via advertisements in the local community. Three of them were excluded due to incidental findings in MRI (n = 2, hydrocephalus and multiple sclerosis) and teeth brace (n = 1). No participant was determined to have excessive movement artefacts. 20 participants (mean age 17.6 ± 1.3 years) were included for the final data analysis. The subjects were generally healthy, with no acute or chronic somatic or functional disease, neither a history of head trauma or fainting. Their body mass index (BMI) was normal (mean BMI 21.1 ± 2.4), and they did not have current or lifetime histories of any major psychiatric diagnoses, including eating disorders, schizophrenia or other psychotic disorders. The participants were MRI compatible, with no metal implants in the body (e.g., pacemakers, surgical devices), no phobic anxiety, claustrophobia, ADHD nor pregnancy. They did not meet any fMRI exclusion criteria, like evidence of structural brain abnormality on the structural MRI scan. All of them were female, 17 right-handed (85%) and 3 left-handed (15%). Participants were scanned satiated and hydrated.

### MRI measurements and analysis

For the MR imaging a 3.0 Tesla (T) scanner (Magnetom Verio, Siemens, Erlangen, Germany) with a standard 12-channel head coil was used. T1-weighted magnetization prepared rapid acquisition gradient echo (MPRAGE) sequence parameters were set to TR = 1950 ms, TE = 3.30 ms, flip angle = 9°, slice thickness = 1.0 mm, TA = 4:38, voxel size = 0.9 × 0.7 × 1.0 mm^3^ and field of view = 220 × 178 mm^2^. EPI (echo planar imaging) BOLD (blood oxygenation level dependent) fMRI sequence parameters were set to TR = 2400 ms, TE = 30 ms, flip angle = 90°, field of view = 220 × 220 mm^2^; voxel size = 2.3 × 2.3 × 3.0 mm^3^ and acquisition time of 7:06, the fMRI paradigm covered 174 measurements.

Image pre-processing and statistical analyses were done with software SPM12 (Wellcome Trust Centre for Neuroimaging, London, UK) while running MATLAB (R2019a; MathWorks, Natick, MA, USA). Prior to statistical analysis, images were realigned to the mean, normalized to a standard EPI-template and finally smoothed with an isotropic Gaussian kernel of 8 mm. Data were also subjected to high-pass filtering (cut-off period: 128 s), low-pass filtering with the hemodynamic response function (HRF) and correction for temporal autocorrelations (based on a first-order autoregressive model). Analysis was performed using a general linear model approach. Normal distribution was verified with the Kolmogorov–Smirnov test. For each subject, the first level design matrix including ‘food condition’ was calculated. After model estimation, the resulting first-level contrast images from each subject were used for second-level analysis, treating individual subjects as a random factor and including Bonferroni correction to counteract the multiple comparisons problem. At the group level, we performed two-sample t-tests to address group differences between high- and low-calorie food. In all analyses, an initial threshold of *p* < 0.05 (corrected, FWE- familywise error rate) was used. Extend threshold was k = 10 voxels. As the blood oxygenation differences change relatively slow, the peak response is approximately 5 s after the brain activation [33], it was considered in statistical fMRI analysis. Motion correction was accomplished using an intra-run realignment algorithm with the first image used as a reference. Data was examined regarding movement artefacts, with thresholds for excessive movement < 2.3° in angular deviation, and less than one voxel in translational deviation, as it is usually performed [34].

### Stimulation paradigm

**Fig. 1:**
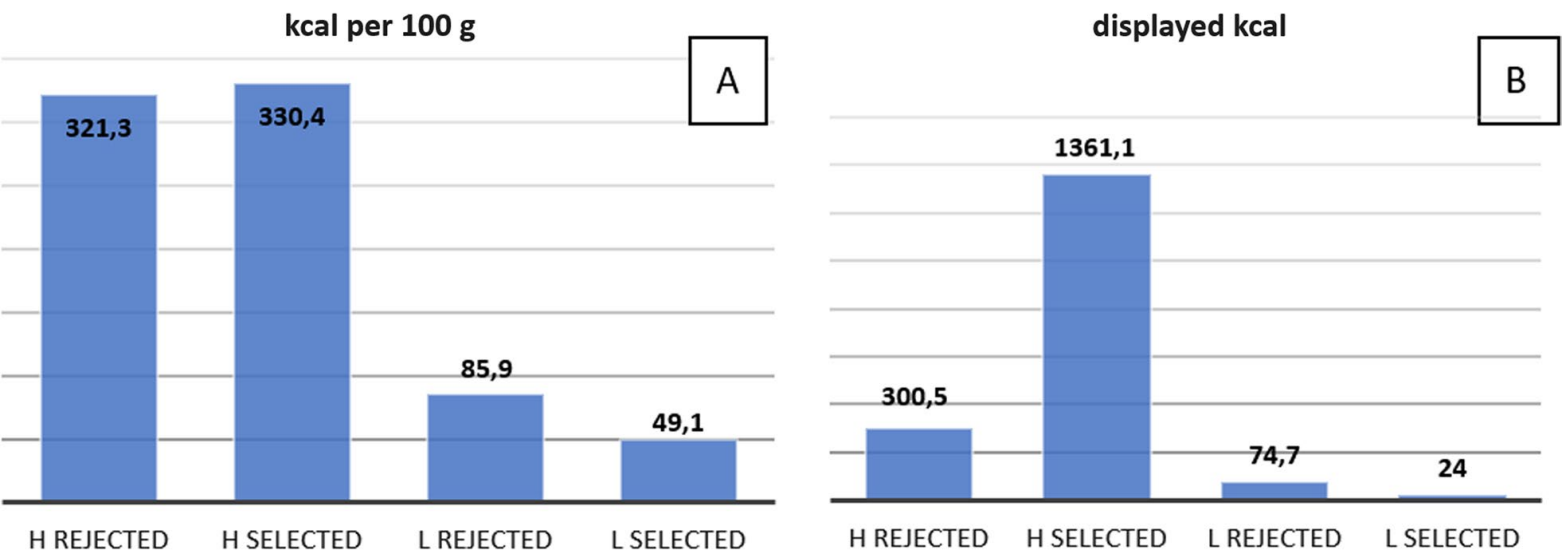
**A** Average of calorie amount per 100 g in each group of images. **B** Average of the calorie amount displayed on the picture. H rejected: high-calorie food pictures, rejected by the group of patients; H selected: high-calorie food pictures, selected by the group of patients; L rejected: low-calorie food pictures, rejected by the group of patients; L selected: low-calorie food pictures, selected by the group of patients

We have established a database consisting of 106 food pictures (1024 × 768 pixels) taken by our team with a high resolution camera. Photos were divided into two groups, according to the calorific value: 52 pictures of high-calorie food and 54 of low-calorie food. Calorie content was taken upon the information from the meal producer, or from the NHS calorie checker, created by British National Health Service to calculate calorie intake [35]. Calorific values of all photographed meals are presented in the additional file (see Additional file 1). In some cases, there was a noticeable difference in characteristics of the given product, i.e. calorific value per 100 g may vary to estimated number of kcal presented on the picture, due to the viewed amount of the meal (e.g., crispbread has 340 kcal per 100 g, but one displayed slice has only 35 kcal). Meals were presented on a light gray background, appearing ready to consume. Evaluation of all pictures was performed by a group of patients diagnosed with AN in matching age and sex, who were not included in this fMRI study, the latter only consisting of healthy participants. AN patients selected 21 images of the most and 21 images of the least willingly eaten meals. Although they did not know the exact calorie contents, they chose intuitively photos that were assigned with the highest and the lowest calorie ratings (Fig. 1).

**Fig. 2:**
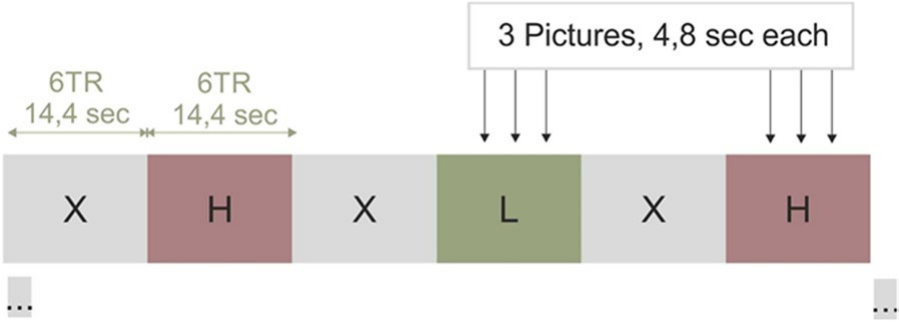
Block-design paradigm. H—high-calorie food pictures; L—low-calorie food pictures; X—fixation cross

The experiment was designed as a block fMRI with conditions of randomly altering high- and low-calorie food images and an image of a fixation cross (Fig. 2).

**Fig. 3:**
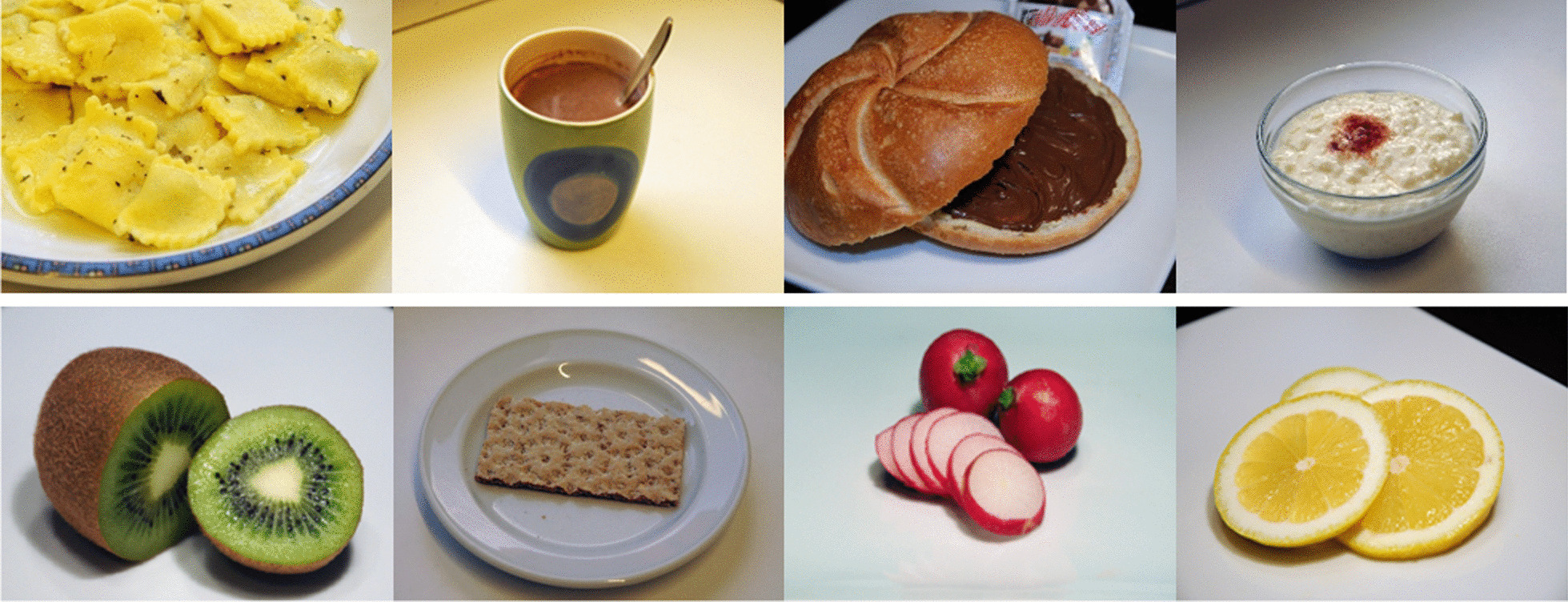
Examples of high- and low-calorie food pictures

The duration of each condition lasted 14.4 s. Each visual activation task consists of 3 pictures from the same group according to calorific value. High-calorie food images included meals like French fries, sweets or hamburger. As low-calorie food pictures served those of fresh vegetables and fruits or processed meals like rice (examples given in the Fig. 3).

A centered fixation cross was used as a baseline condition, which represents a visual stimulus without food context. In total, the paradigm consists of 29 blocks, i.e. 7 blocks with 3 pictures from each category regarding calorific value, respectively 21 images with high- and 21 images with low-calorie content (TA: 6 min 58 s). Images were displayed via MRI-compatible visual system (goggles, NordicNeuroLab), downsized to ~ 230 KB to match the required technical features of the goggles. The visual system is equipped with an additional vision correction function, which enabled us to incorporate also participants with slight visual impairment (+ 2 to − 5 diopter).

To evaluate the visual food stimuli paradigm, we have compared brain reactions after applying different contrasts—high-calorie food pictures versus baseline (*H *vs.* X*), low-calorie food pictures versus baseline (*L *vs.* X*), high- versus low-calorie food pictures (*H *vs.* L*). Unique reception of high-calorie food stimuli was already described in patients diagnosed with AN, comparing to healthy controls [17, 36]. We hypothesized, that by applying the contrast *H *versus* L* on the group of patients diagnosed with AN, their cerebral responses to both types of food will be distinguished, and thus the specific response to the symptom-provoking high-calorie stimulus will be emphasized. Hence, as a first step, we hereby present the analysis of these contrasts on healthy participants.

## Results

**Fig. 4:**
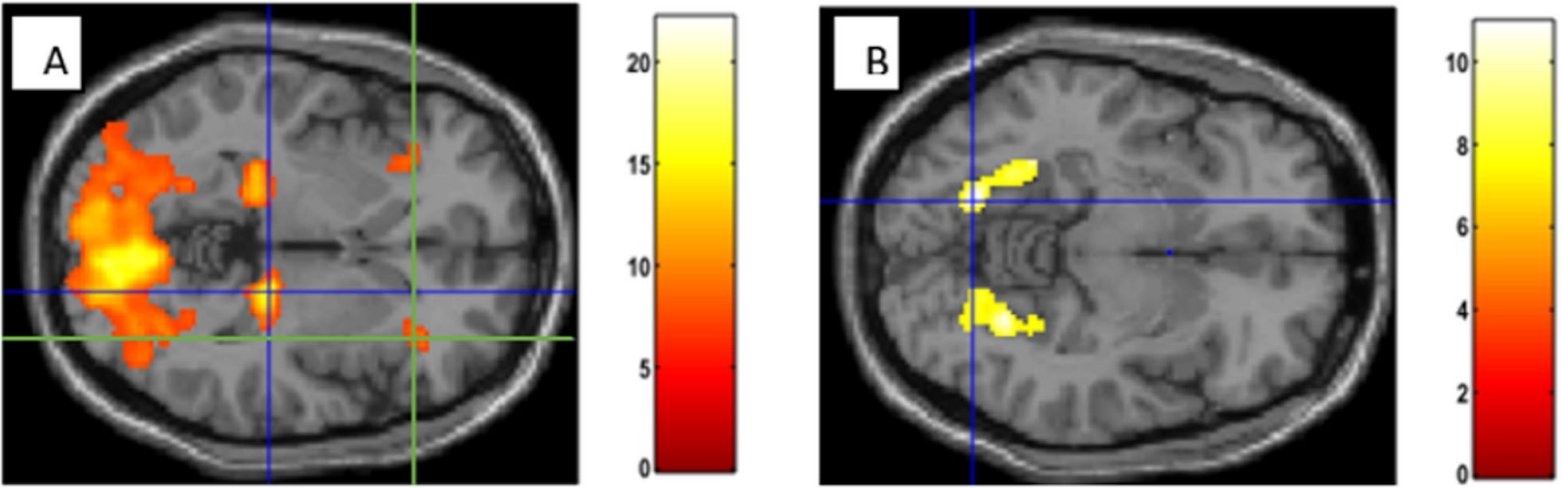
**A** Regions of increased BOLD signal detected in response to high-calorie food images versus baseline contrast. **B** Regions of increased BOLD signal detected in response to high- versus low-calorie food images contrast. **A** The blue cross is centered on the thalamus, the green cross is centered on the insula (*p* = .000, FWE). Both regions were activated bilaterally, but exemplary displayed here only in one hemisphere. **B** Bilateral fusiform gyrus (*p* = .000, FWE)

Due to high-calorie food pictures (*H *vs.* X*), increased blood-oxygen-level-dependent signal (BOLD) was detected within the visual cortex (including the cuneus, fusiform gyri and angular gyri), thalami (Fig. 4A), insulae (Fig. 4A), the right dorsolateral prefrontal cortex (DLPFC), the Broca´s area, the left amygdala, the left putamen and bilaterally in the premotor cortex and the supplementary motor area (*p* < 0.05) (Table 1).

**Table 1:** Regions of increased BOLD contrast in response to high-calorie food images compared to the baseline

MNI coordinates	Anatomic region	*p*-value	k	peak T-value
− 12, − 98, 24	Left secondary visual cortex, cuneus, fusiform gyrus (similar area on the right side)	.000, FWE	15,401	22.10
18, − 26, − 2	Right thalamus	.000, FWE	215	15.69
− 24, − 30, − 2	Left thalamus	.000, FWE	211	12.30
36, 32, 0	Right insula	.000, FWE	125	11.36
50, 34, 30	Right DLPFC	.000, FWE	33	10.04
− 56, 20, 12	Broca´s area	.000, FWE	48	9.60
12, 56, 40	Right DLPFC	.000, FWE	26	9.43
− 30, 28, 0	Left insula	.000, FWE	63	9.17
− 44, 2, 42	Left premotor cortex, medial frontal gyrus	.000, FWE	53	8.91
− 26, − 4, − 20	Left amygdala	.000, FWE	32	8.72
− 30, − 16, − 6	Left putamen	< .001, FWE	11	8.51
− 10, 14, 52	Supplementary motor area	.000, FWE	166	8.48
48, 8, 32	Right premotor cortex, medial frontal gyrus	.000, FWE	29	8.45

Extend threshold for the cluster size was k = 10 voxels

*BOLD* Blood oxygenation level dependent; *MNI* Montreal Neurological Institute; *DLPFC* Dorsolateral prefrontal cortex; *FWE* Familywise error rate

Neural activity due to low-calorie food pictures (*L *vs.* X*) showed stronger activity in the visual area (also including fusiform gyri and angular gyri), the right precentral gyrus, the right temporal pole, the left opercular part of inferior frontal gyrus (Broca´s area), the left insula, the left hippocampus, the left parahippocampal gyrus, bilaterally premotor cortex and thalami (*p* < 0.05) (Table 2).

**Table 2:** Regions of increased BOLD contrast in response to low-calorie food images compared to the baseline

MNI coordinates	Anatomic region	*p*-value	k	peak T-value
10, − 74, − 6	Secondary visual cortex, cuneus, fusiform gyrus	.000, FWE	12,482	17.34
− 48, 12, 22	Left opercular part of inferior frontal gyrus. Broca’s area	.000, FWE	144	11.66
20, − 28, − 2	Right thalamus	.000, FWE	70	10.51
42, 26, − 18	Right temporal pole	.000, FWE	62	10.36
62, − 8, 26	Right precentral gyrus	.000, FWE	26	9.98
− 34, − 6, 12	Left insula	.000, FWE	16	9.89
− 18,− 30, 0	Left thalamus	.000, FWE	87	9.73
− 32, − 8, − 18	Left hippocampus	.000, FWE	26	9.6
− 28, − 4, − 40	Left parahippocampal gyrus	.000, FWE	14	8.54
26, − 72, 42	Right superior parietal lobule (precuneus)	.000, FWE	14	8.46
40, 2, 24	Right premotor cortex (middle frontal gyrus)	< .001, FWE	10	8.37
− 42, − 2, 44	Left premotor cortex (middle frontal gyrus)	< .001, FWE	12	8.09
− 10, 6, 64	Left premotor cortex (superior frontal gyrus)	< .001, FWE	26	8.06

Extend threshold for the cluster size was k = 10 voxels

*BOLD* Blood oxygenation level dependent; *MNI* Montreal Neurological Institute; *FWE* Familywise error rate

Comparing the neural activation when looking at high-calorie food images versus baseline with low-calorie food pictures versus baseline within the visual cortex, there was stronger response detected regarding *H *versus* X* contrast—with extended number of activated voxels and higher T-value. Similar activation could be found in the thalami and the premotor cortex. On the contrary, a larger area with higher T-value was identified within Broca´s area when comparing low-calorie food images versus baseline to high-calorie food images versus baseline. Increased BOLD signal was detected in the left insula due to both contrasts (*H *vs.* X* and *L *vs.* X*), but with stronger T-value and an extended number of involved voxels regarding high-calorie stimuli versus baseline contrast. The right insula was activated only when analysing *H *versus* X* contrast (FWE). The right DLPFC, the left amygdala and the left putamen were activated significantly also only in response to high-calorie stimuli versus baseline contrast (*H *vs.* X*, FWE). The left hippocampus and the left parahippocampal gyrus, the right temporal pole, the right precentral gyrus, the right superior parietal lobule (precuneus) were activated when applying low-calorie stimuli versus baseline contrast (*L *vs.* X*, FWE).

Analysing the comparison of high- versus low-calorie food led to bilateral enhancement of BOLD signal in primary, secondary and associative visual cortices (including fusiform gyri—Fig. 4 B) and the angular gyri (*p* < 0.05) (Table 3).

**Table 3:** Regions of increased BOLD contrast in response to high-calorie food compared to low-calorie food images

MNI coordinates	Anatomic region	*p*-value	k	peak T-value
− 18, − 68, − 8	Bilateral secondary visual cortex, fusiform gyrus	.000, FWE	302	10.94
− 34, − 80, 18	Left associative visual cortex, cuneus	.000, FWE	128	10.75
40, − 76, 16	Right associative visual cortex, cuneus	.000, FWE	39	8.01
14, − 78, 8	Bilateral primary visual cortex, cuneus	.000, FWE	1751	10.62
34, − 66, 26	Right angular gyrus (similar area on the left side)	.000, FWE	17	8.76
24, − 28, 0	Right thalamus	< .024, uncorr	91	6.04
38, 30, 4	Right insula	< .038, uncorr	75	5.58
− 24, − 28, − 24	Left fusiform gyrus (similar area on the right side)	< .157, uncorr	32	4.95
− 30, 30, − 8	Left insula	< .121, uncorr	39	4.81
− 22, − 28, − 4	Left thalamus	< .140, uncorr	35	4.69
− 8, 34, − 20	Left orbitofrontal area	< .363, uncorr	13	3.82

Extend threshold for the cluster size was k = 10 voxels

*BOLD* Blood oxygenation level dependent; *MNI* Montreal Neurological Institute; *FWE* Familywise error rate

## Discussion

To establish a reliable base of image dataset, all food pictures were rated in advance by a group of patients with anorexia nervosa. This patient group is considered to be extremely cautious of potentially fattening meals and reacts specifically toward the latter [17, 36, 37] due to the nature of AN [5]. In our study, the group of patients with AN reacted in line with those assumptions, clearly choosing, among all pictures, those with the highest calorie amount displayed (Fig. 1, Additional file 1). Only food images with the highest index of unequivocalness were used in the study. Images included the full range of food products: processed and raw, solid and fluid, with all 5 basic tastes (sweet, salty, bitter, sour and umami). To gain full coverage of different complexity status we have included pictures of single subjects (e.g., leaf of lettuce), several items (e.g., few radishes) and complicated meals (e.g., hamburger), as suggested by Blechert et al. [15].

By meticulously crafting the dataset of visual food stimuli, we were able to obtain results similar to other studies. Among regions activated in food images processing, the most common results indicated bilateral posterior fusiform gyrus, the left lateral orbitofrontal cortex (OFC) and the left middle insula [38]. We have also received enhanced activity of the fusiform gyrus due to visual presentation of all food images—both high- and low-calorie (*p* = 0.000, FWE). Interestingly, signal enhancement in fusiform gyrus was significantly higher due to high- against low-calorie stimuli (*H *vs.* L*; *p* = 0.000, FWE) (Fig. 4B). This could be explained by the fact, that the fusiform gyrus was activated specifically during watching emotional pictures [39]—and palatable food can be considered as a vital positive stimulus in healthy teenage participants. Furthermore, it can also be assumed that stimuli rated in advance by a group of patients diagnosed with AN could be specifically assigned to the emotional processing of food images, as they have been chosen as the most and the least willingly eaten food. Although both contrasts (*H *vs.* X* and *L *vs.* X*) led to a stronger response in the fusiform gyrus, only the application of the contrast (*H *vs.* L*) showed a significant difference in neural activation in this brain region.

The insula is a region involved in appetite regulation, with well documented increased BOLD signal upon the presentation of food stimuli [18, 20, 22–24, 36]. Insular activity was positively correlated in state of hunger with pleasantness of high-calorie food images [21, 25], as well as with appetite rating (in the right hemisphere) [21]. And, as expected, we received increased insular activity due to high-calorie food stimuli (Fig. 4A). This highly statistically significant enhancement of insular signal may also be an age-related feature, as a similar group of healthy adolescents (mean age 16 years) presented higher activation of insula than adults [26].

We observed an enhancement of signal in the thalamus, when watching food images (*p* = 0.000, FWE) (Fig. 4A), as well as comparing high-calorie food images to low-calorie (on the right side p_uncorr._ < 0.024, on the left side p_uncorr._ < 0.140). As an activation of the thalamus was also found in other studies on children [11, 40] but not in adults, there is an assumption of age-related decreases in activity [11].

Furthermore, the presented study shows significantly increased response in dorsolateral prefrontal cortex when comparing brain activity to high-calorie food images to baseline (*H *vs.* X*; *p* = 0.000, FWE). An activation of DLPFC was correlated with successfully executing dietary self-control and might be involved in choosing long-term over short-term decisions [41], i.e. considering most delicious, but also fattening meals. It is in line with our results, when displaying low-calorie food images comparing to baseline (*L *vs.* X*), we have found enhancement of signal in DLPFC, however, the difference was not highly significant. That is why we didn´t obtain increased activity of DLPFC after applying the *H *versus* L* contrast.

High-calorie food images seem to be more captivating than low-calorie food images in adolescents. The visual processing cortex of adolescents is more sensitive to high-calorie food images, compared to adults [11]. This could be because of greater importance of high-calorie food intake during the growing period of juvenility. It was examined, that children react specifically (more emotionally) to high-energy-dense food images [42] which could be compared to our high-calorie food images. The children’s reaction to low-energy-dense food was depending on the feeling of fullness. They have preferred high-energy–density food over low, and the latter over office supplies (e.g., markers, paper clips) [42]. Children preferred larger portions of high-energy-dense food and smaller of low-energy-dense food [43]. These results are consistent with ours, when the rating group (AN) ranked the low-calorie food images with the smaller calorie amount presented on the picture as more pleasant (Additional file 1, Fig. 1). Furthermore, obtaining a seemingly contradictory result in our study for high-calorie food images—indicating larger portions as the least attractive—is probably due to the characteristics of our rating group diagnosed with AN.

Application of the contrast *H *versus* L* to a future group of patients with anorexia nervosa may provide more accurate results as tailored to meet specific disease trigger features. Although, previous studies revealed increased activity in fusiform gyrus arisen in response to high-calorie food photos, contrasted to homogeneous, neutral pictures (e.g., bricks or stones) [18], in-depth post factum analysis has evolved the hypothesis, that the obtained results may be also due to the coincidence of the involvement of the higher visual cortex, in case more complex stimuli are contrasted with simpler ones. When comparing visual stimuli of different complexity level (e.g., in terms of variety, repeatability, number of elements, colors, background, but also the importance of the presented object—like images of faces or a simple pattern), there might be a risk of falsifying the results. That is why it is essential to properly create a paradigm and choose the right contrast for the research hypothesis, and then "de-code" the paradigm in order to analyse the meaning of the results obtained.

### Outlook and limitations

We developed and evaluated a new setup of visual stimuli incorporating customized food images for a block-designed fMRI study on a healthy adolescent population group. We are planning the clinical implementation of our paradigm including adolescent female patients diagnosed with anorexia nervosa (AN), as its onset occurs typically in adolescence. Patients diagnosed with AN are vulnerable to high-calorie meals pictures, reacting to them emotionally and cognitively [17], therefore it can be assumed that they will show differences in cerebral activity compared to healthy participants. We hypothesize, that the use of *H *versus* L* contrast will lead to statistically higher significant differences between healthy participants and patients with AN, as the latter respond differently to high-calorie food stimuli, but not the low-calorie food [17]. It would be also worth to include males in our comparison, as according to Luo et al. [40] healthy boys displayed greater brain responses to food versus non-food cues in visual cortex and right hippocampus. It is unclear, whether the percentage of male patients with AN differs according to age—a greater prevalence of boys is described in younger patients [44], however, also no difference in prevalence by age [45]. Additionally, there is a growing number of publications [44, 46] which show, that AN affects also younger children, therefore it would be interesting to broaden the groups variability with younger children, as well as with adults. Shorter disease duration and younger age of the onset are associated with predictors of better outcomes (45, 47), so a thorough understanding of the mechanisms underlying anorexia nervosa seems crucial.

As a main limitation we find a restricted number of participants included in our analysis (n = 20). This was partly due to drop-outs, as well as the timeline of the study. The current study is a preliminary study to evaluate our paradigm, which will then be implemented within a study on a group of participants with AN.

## Conclusions

Although fMRI studies including visual food paradigms are becoming more and more popular, there is a lack of golden standards regarding creating a flawless study design. In our study we found that brain activity due to visual stimuli of well-characterized types of food showed differences regarding high or low calorific values. We could reveal, that applying various contrasts allows to highlight significant features in the paradigm and to examine individual differences in food-cue responsivity. We hypothesize, that choosing the contrast high- versus low-calorie food stimuli is crucial in analysis performed on healthy population and will be even more essential on patients diagnosed with anorexia nervosa. A possible disadvantage of using this contrast might be omission of some results, due to lower statistical power.

## Supplementary Information

**Additional file 1.** Calorific values of all photographed meals

## Data Availability

The datasets generated and/or analysed during the current study are not publicly available due to participant confidentiality and the commitment given to all participants in protecting their identity. Data are available de-identified from the corresponding author on reasonable request.

## Abbreviations

ADHD: Attention deficit hyperactivity disorder
AN: Anorexia nervosa
BMI: Body mass index
BOLD: Blood-oxygen-level-dependent signal
DLPFC: Dorsolateral prefrontal cortex
ED: Eating disorders
EPI: Echo planar imaging
fMRI: Functional magnetic resonance imaging
FWE: Familywise error rate
HRF: Hemodynamic response function
H vs. L: High- versus low-calorie food visual stimuli
H vs. X: High-calorie food visual stimuli versus baseline
kcal: Kilocalorie
L vs. X: Low-calorie food visual stimuli versus baseline
MPRAGE: Magnetization prepared rapid acquisition gradient echo
MRI: Magnetic resonance imaging
NHS: National Health Service
OFC: Orbitofrontal cortex
T: Tesla
TA: Acquisition time
TE: Echo time
TR: Repetition time
Uncorr.: Uncorrected
