# Supplementary material for: Evaluation of visual food stimuli paradigms on healthy adolescents for future use in fMRI studies in anorexia nervosa
Source: J Eat Disord. 2023 Mar 6;11:35. doi: 10.1186/s40337-023-00761-8 (PMC9987124; doi:10.1186/s40337-023-00761-8)
Supplement: Supplementary file 1 — Additional file 1. Calorific values of all photographed meals [file 40337_2023_761_MOESM1_ESM.docx]

Calorific values of all photographed meals.

| Name of the meal | Kcal per 100 g | Estimated weight of the product/s (g) | Estimated number of kcal presented on the picture |
| --- | --- | --- | --- |
| **High-calorie food images included in the fMRI paradigm** | | | |
| Burger 1 | 262.1 | 115.8 | 303.5 |
| Butter 2 | 743 | 250 | 1857.5 |
| Fried squid | 203.4 | 235 | 478 |
| Chips 2 | 528 | 140 | 739.2 |
| Ice cream 2 | 198.2 | 110 | 218 |
| Ice cream 3 | 194.9 | 136 | 265 |
| Children’s dairy snack | 92 | 300 | 276 |
| Kakao (g) | 77 | 249.4 | 192 |
| Lasagne | 135 | 400 | 540 |
| Rice pudding | 370 | 54 | 200 |
| MMs | 481 | 453.6 | 2181.8 |
| Muffin | 455.5 | 675 | 3075 |
| Pasta casserole | 305 | 200 | 610 |
| Pasta 3 | 192 | 250 | 480 |
| Roll with chocolate spread | 324.6 | 65 | 211 |
| Nutrition drinks 1 (g) | 150 | 9296 | 13944 |
| Pizza 1 | 266.4 | 107 | 285 |
| French fries 2 | 290 | 240 | 696 |
| Bar of chocolate 2 | 584 | 100 | 584 |
| Chocolate candy bar | 510.3 | 58 | 296 |
| Birthday cake | 576 | 200 | 1152 |
| **High-calorie food images NOT included in the fMRI paradigm** | | | |
| Supper | 106.8 | 844.7 | 902 |
| Spread | 255 | 25 | 63.75 |
| Diet margarine | 540 | 10 | 54 |
| Burger 2 | 281 | 171 | 480 |
| Burger 3 | 215 | 121 | 260 |
| Butter | 743 | 10 | 74.3 |
| Chicken nuggets | 249 | 71.3 | 178 |
| Chips | 536 | 25 | 134 |
| Dressing 1 | 178 | 25 | 44.5 |
| Dressing 2 | 286 | 25 | 71.5 |
| Ice creams 1 | 172.5 | 560 | 966 |
| Strawberry yogurt cake | 219 | 180 | 394 |
| Breakfast | 157.9 | 460.1 | 726.3 |
| Cheese spread | 232 | 20 | 46.4 |
| Cookies | 400 | 30 | 120 |
| Pasta 1 | 369 | 58 | 214 |
| Pasta 2 | 180 | 250 | 450 |
| Nuts | 580 | 80 | 464 |
| Chocolate spread | 533.3 | 15 | 80 |
| Nutrition drinks 2 (g) | 150 | 464.8 | 697.2 |
| Fruit yogurt | 122.4 | 125 | 153 |
| Pizza 2 | 131.1 | 270 | 354 |
| French fries 1 | 290 | 80 | 232 |
| Popcorn 1 | 427 | 100 | 427 |
| Popcorn 2 | 525 | 12 | 63 |
| Sponge cake with jam | 284 | 50 | 142 |
| Bar of chocolate | 566 | 50 | 283 |
| Bun | 262 | 50 | 131 |
| Soy vanilla pudding | 80 | 125 | 100 |
| Spaghetti Bolognese | 241.9 | 270 | 653 |
| Wallnuts | 650 | 20 | 130 |
| **Low-calorie food images included in the fMRI paradigm** | | | |
| Apple 2 | 40 | 18.75 | 7.5 |
| Berries 4 | 42.1 | 122 | 51.4 |
| Broccoli1 | 23.3 | 60 | 14 |
| Strawberries 1 | 25 | 24 | 6 |
| Yogurt 1 | 45 | 250 | 112.5 |
| Yogurt2 | 50 | 115 | 57.5 |
| Carrots 2 | 33.3 | 30 | 10 |
| Kiwi | 56 | 75 | 42 |
| Crispbread 2 | 340 | 10.3 | 35 |
| Limes 2 | 47.9 | 16.3 | 7.8 |
| Orange | 36 | 65 | 23.4 |
| Parsley | 36.3 | 8 | 2.9 |
| Radish | 12.5 | 34.3 | 4.3 |
| Rice | 131.4 | 70 | 92 |
| Rocket lettuce | 10 | 10 | 1 |
| Leaf of salat | 10 | 7 | 0.7 |
| Sliced cucumber | 15 | 24 | 3.6 |
| Tomatos 3 | 11.8 | 80 | 9.4 |
| Lemons | 15 | 40 | 6 |
| Zucchini | 18 | 98 | 17.64 |
| Snow pea pods | 34 | 0.6 | 0.2 |
| **Low-calorie food images NOT included in the fMRI paradigm** | | | |
| Apple 1 | 40 | 37.5 | 15 |
| Berries | 49.3 | 232 | 114.4 |
| Berries 2 | 56.25 | 112 | 63 |
| Berries 3 | 57.5 | 72 | 41.4 |
| Broccoli 2 | 23.3 | 80 | 18.64 |
| Mushrooms | 9 | 56 | 5.04 |
| Strawberry cream | 33 | 100 | 33 |
| Strawberries 2 | 25 | 30 | 7.5 |
| Fish with sauce, rice and carrots | 130.5 | 190 | 248 |
| Fish 1 | 76 | 68 | 51.7 |
| Fish 2 | 181.25 | 80 | 145 |
| Fruit yogurt | 75 | 100 | 75 |
| Oatmeal | 370 | 46 | 170 |
| Cup of yogurt with fruits | 50.9 | 139.4 | 71 |
| Carrots 1 | 27.5 | 80 | 22 |
| Crispbread 1 | 380 | 14.8 | 56 |
| Kohlrabi | 23 | 200 | 46 |
| King oyster mushrooms 1 | 35 | 300 | 105 |
| King oyster mushrooms 2 | 35 | 100 | 35 |
| Limes 1 | 48 | 64 | 30.72 |
| Corn | 80 | 33.4 | 26.7 |
| Tangerines | 58 | 138 | 80 |
| Noodle soup | 34 | 250 | 85 |
| Paprika | 26 | 150 | 39 |
| Rice waffles | 372 | 17 | 63.24 |
| Salad 1 | 56.4 | 87 | 49 |
| Celery | 26 | 54.2 | 14.1 |
| Pole beans | 21.7 | 50 | 10.8 |
| Soup 1 (g) | 35 | 216 | 76 |
| Sushi 1 | 150.5 | 248.1 | 373.4 |
| Tomatos 1 | 20 | 90 | 18 |
| Tomatos 2 | 20 | 75 | 15 |
| Whole wheat bread | 210 | 125.7 | 264 |
